# Image-Processing Software for High-Throughput Quantification of Colony Luminescence

**DOI:** 10.1128/mSphere.00676-18

**Published:** 2019-01-02

**Authors:** Eyal Dafni, Iddo Weiner, Noam Shahar, Tamir Tuller, Iftach Yacoby

**Affiliations:** a School of Plant Sciences and Food Security, The George S. Wise Faculty of Life Sciences, Tel Aviv University, Tel Aviv, Israel; b Department of Biomedical Engineering, The Iby and Aladar Fleischman Faculty of Engineering, Tel Aviv University, Tel Aviv, Israel; c The Sagol School of Neuroscience, Tel Aviv University, Tel Aviv, Israel; University of Iowa

**Keywords:** fluorescent-image analysis, microbial method, software

## Abstract

Many microbiological assays include colonies that produce a luminescent or fluorescent (here generalized as “luminescent”) signal, often in the form of luminescent halos around the colonies. These signals are used as reporters for a trait of interest; therefore, exact measurements of the luminescence are often desired. However, there is currently a lack of high-throughput methods for analyzing these assays, as common automatic image analysis tools are unsuitable for identifying these halos in the presence of the inherent biological noise. In this work, we have developed CFQuant—automatic, high-throughput software for the analysis of images from colony luminescence assays. CFQuant overcomes the problems of automatic identification by relying on the luminescence halo's expected shape and provides measurements of several features of the colonies and halos. We examined the performance of CFQuant using one such colony luminescence assay, where we achieved a high correlation (*R* = 0.85) between the measurements of CFQuant and known protein expression levels. This demonstrates CFQuant's potential as a fast and reliable tool for analysis of colony luminescence assays.

**IMPORTANCE** Luminescent markers are widely used as reporters for various biologically interesting traits. In colony luminescence assays, the levels of luminescence around each colony can be used to compare the levels of traits of interest for different strains, treatments, etc., using quantitative measurements of the luminescence. However, automatic methods of obtaining this data are underdeveloped, making this a laborious manual process, especially in analyzing large numbers of colonies. The significance of this work is in developing an automatic, high-throughput tool for quantitative analysis of colony luminescence assays, which will allow fast collection of qualitative data from these assays and thus increase their overall usability.

## Abstract (precis)

Luminescent markers are widely used as reporters for various biologically interesting traits. In colony luminescence assays, the levels of luminescence around each colony can be used to compare the levels of traits of interest for different strains, treatments, etc., using quantitative measurements of the luminescence. However, automatic methods of obtaining this data are underdeveloped, making this a laborious manual process, especially in analyzing large numbers of colonies. The significance of this work is in developing an automatic, high-throughput tool for quantitative analysis of colony luminescence assays, which will allow fast collection of qualitative data from these assays and thus increase their overall usability.

## INTRODUCTION

Luminescent and fluorescent reporters (here generalized as “luminescent” reporters [1]) are widely used in all fields of biological research (2–5). As research progresses, the scale of experiments is growing and the need for high-throughput quantitative analyses from a large set of luminescence-based assays is rising accordingly (6).

The analysis of colony luminescence is a major step in various assays; in such setups, colonies typically express a luminescent marker (e.g., green fluorescent protein [GFP], luciferase, mCherry, etc.) which is used to verify transformation (7–9), report on gene expression (10–13), report on cellular localization (14–16), indicate binding between entities (17–19), or monitor growth rates (20–22) or for any other purpose. In these types of assays, the output usually consists of a pair of images: (i) an image displaying the plated microbe or cell colonies and (ii) an image of the same plate taken with a relevant camera and exhibiting various levels of colony luminescence. The typical task is that of quantifying the probed trait from the luminescence image while normalizing the signal by the size of the colonies, as deduced from the first image.

**FIG 1:**
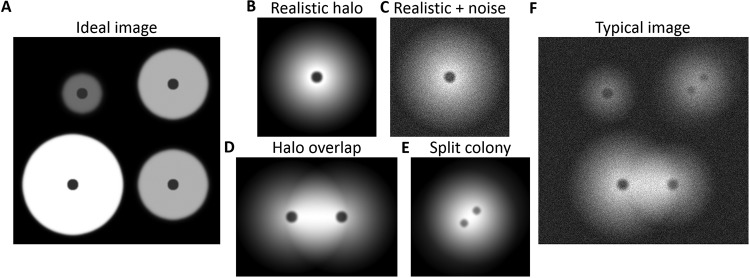
Illustration of common artifacts in automatic identification of luminescent halos. (A) An ideal image, with clear halo borders. The darker spots in the center of each halo represent the colonies. (B) A more realistic halo, with its values slowly decreasing from the center. This creates soft edges, with very small difference in values between the halo's edge and the background. (C) As described for panel B, but with the addition of noise, which lessens the difference between the halo and the background even further. (D) Two overlapping halos. Notice that the area of overlap is brighter (i.e., has higher values) than the other side of each halo. (E) A split colony, separated into two areas (indicated by the two dark spots). (F) The common result of a colony luminescence assay: the image is noisy, the halo values are gradually decreasing, and both overlaps and split colonies may exist.

Current technologies for large-scale accurate quantification of these images are underdeveloped. The reason for this gap in relevant analysis tools lies in the inherent noise of these biological assays. While a theoretical plate (Fig. 1A) could be analyzed with common image-processing tools (e.g., the ImageJ [23] user interface or similar tools), images obtained from real experiments contain artifacts which hinder the ability of general image-processing algorithms to properly analyze them. The most common artifacts are soft edges of the luminescent halos (Fig. 1B), image noise (Fig. 1C), halo overlaps (Fig. 1D), and split colonies (Fig. 1E). If general image-processing tools are used, quantitative analysis of images with combinations of all these artifacts (Fig. 1F) requires heavy user input. Thus, researchers interested in accurate quantification of their assay often have to (i) adjust the contrast until a threshold that seems right according to their perception is found, (ii) manually mark each halo separately, and (iii) manually subtract halo intensity data resulting from overlapping regions. The laboriousness of this procedure drives researchers toward either keeping their assays small or analyzing the results qualitatively.

To address these issues, we developed a general software package—CFQuant—for analyzing colony luminescence while taking typical levels of biological noise (Fig. 1) into consideration. As a study case, we used the Rhodobacter capsulatus high-throughput screening system (24, 25). In that assay, plates containing algal colonies are overlaid with engineered bacteria which produce GFP in the presence of gaseous hydrogen (H_2_). This system, which generates a luminescence image (GFP) alongside a colony image (chlorophyll), is typically used as a qualitative phenotypic screen that reports on desirable genetic traits in heterogeneous populations (25–29). This assay represents a classical large-scale experiment in which the output is a colony luminescence image with an array of biological noise data which have so far prevented a quantitative analysis. Using our novel image-processing tool, we show here that we are able to overcome the noise issues and formulate a sound quantitative prediction of active-enzyme abundance in each colony on the basis of these large-scale screening images alone.

CFQuant is available at https://www.energylabtau.com/cfquant.

## RESULTS

### Software details.

Upon initiation of the software, the user is required to upload the colony and halo images and to choose the colony detection method—either arrangement-based or scatter-based detection. To use arrangement-based detection, the user must also upload an approximate arrangement of the colonies in a grid of rows and columns (see Materials and Methods for image requirements). The user also has the choice of either analyzing a single image or performing batch processing—analysis of multiple images—without the user interaction steps.

**FIG 2:**
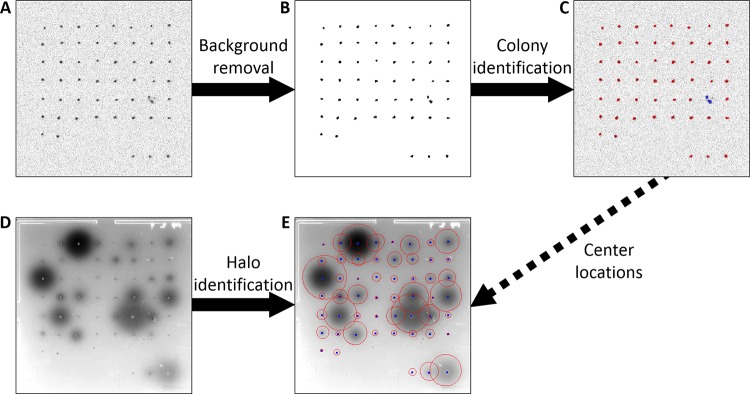
The flow of the software in analyzing a single plate. (A) The colony image of the plate before analysis. (B) The colony image after the background removal step. (C) The colony image after colony identification. The identified colony borders are shown in red. The blue borders mark two areas that were identified as parts of a split colony. (D) The halo image before analysis. Image contrast was adjusted for visibility. (E) The halo image after identification. The identified halo borders are shown in red. The blue dots mark the centers of the colonies, which are used in the halo identification.

Once the input is received, CFQuant starts analyzing the colony image (Fig. 2A). The software begins with an initial background removal step, after which the image is left with several foreground areas (Fig. 2B). However, in some images the number of foreground areas exceeds the specified number of colonies. In arrangement-based detection, CFQuant compares the arrangement of the foreground areas with the user-specified arrangement and determines by this comparison if the excess areas are due to persisting background noise or cases of split colonies (Fig. 1E) or both. It then either joins foreground areas that are in close proximity or deletes low-value ones until no excess areas remain. In scatter-based detection, the colony number is unknown, so the software uses the shapes, sizes, and values of the foreground areas to ensure that background noise is deleted. Split-colony identification is not performed using this method. Regardless of the method chosen, in the final stage the software determines the background threshold value (i.e., the value below which pixels are considered part of the background). Once the colonies are identified, the user can view the results and make changes if necessary (Fig. 2C).

After ensuring that colonies were correctly identified, CFQuant moves on to the halo image (Fig. 2D). For each halo, the software attempts to find the threshold value separating it from the background. Since edge detection is problematic in the luminescent halos (Fig. 1), CFQuant relies on the fact that halos form circular shapes centered at (or near) the middle of the colony. In cases of halo overlaps (Fig. 1D), each halo is expected to resemble a partial circle. Therefore, the software first assigns to each halo the highest possible background threshold value and then gradually decreases it until the area no longer resembles a circle (or a partial one). The stopping point is determined based on the distances corresponding to each edge point from the colony center being roughly equal while still allowing some noise in the distance and center location. As in the previous phase, once all the halos are identified, the user can view the results and make changes if necessary (Fig. 2E).

After the analysis is completed, data on both the colonies and the halos are saved. The saved data include the area, pixel sum, mean pixel value, and maximal pixel value corresponding to each colony and each halo. The latter three parameters are also evaluated using normalized calculations based on the selected background thresholds (see Materials and Methods for more details). In the halo image, pixel values in areas of overlap are corrected to the values expected for each halo to avoid the additive effect in the overlap area (Fig. 1D).

### Automatic identification test.

Colony identification performed using arrangement-based detection correctly identified 100% of the colonies on all 31 plates tested (each plate contained 20 to 109 colonies). Using scatter-based detection on the same plates, 99% of the colonies were correctly identified. In testing performed on plates containing 16 to 656 scattered colonies, 88% of the colonies were correctly identified (83% to 94% for each plate individually). A total of 60 areas representing background noise were mistakenly identified as colonies (3% of identified colonies); all were dark spots at the edge of the plates. Fig. S1 in the supplemental material shows an example of images identified with scatter-based detection.

10.1128/mSphere.00676-18.1 FIG S1 Results of automatic identification of an image with scattered colonies, without manual corrections. (A) Most colonies were correctly identified. However, some noise at the edge of the plate was erroneously identified as well. (B) Halo identification on the same plate. Download FIG S1, TIF file, 0.7 MB. Copyright © 2019 Dafni et al. 2019 Dafni et al. This content is distributed under the terms of the Creative Commons Attribution 4.0 International license.

Among 695 noticeable halos in all 37 plates, 671 (97%) were identified automatically. However, 13% of them were considerably smaller or larger than their size as perceived by eye. Twelve colonies (2% of the identified halos) were erroneously given a halo.

In images with fewer than 200 colonies and sizes of up to 625-by-625 pixels, the durations of automatic identification were at most 5 s for the colony image and 34 s for the halo image, using CFQuant from MATLAB version 2017b on Windows 7 (Intel Core i7 6700; CPU, 3.4 Ghz). It should be noted that the individual images had no more than 36 noticeable halos in them. With the largest colony number (656, only 8 of which had noticeable halos), automatic identification took 21 s for the colonies and 77 s for the halos.

### Performance test.

**FIG 3:**
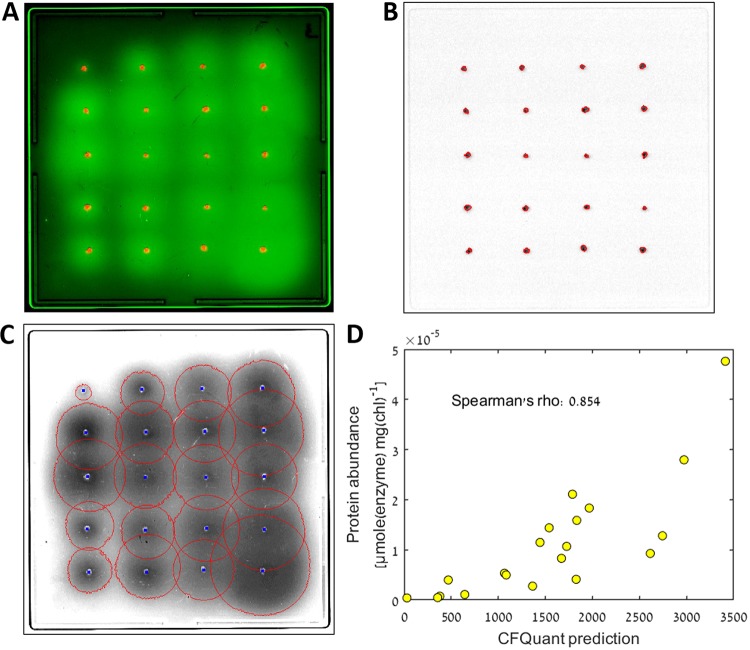
The results of the performance test. (A) Composite image of one of the plates used in the assay, combining the colony and halo images. The colonies are shown in red, and the GFP halos are shown in green. (B) The colony image after CFQuant analysis. The detected colony borders are shown in red. (C) The halo image after CFQuant analysis. The detected borders of the halos are shown in red, and the colony centers are shown in blue. (D) The best correlation of a single halo feature with the known protein expression values of the strains (*P* value < 10*^−^*^5^). The CFQuant prediction represents the normalized sum of pixels for the halos divided by the area of the colonies, as described in the text. The values used in the figure represent averages of the measurements from all four plates. The protein abundance represents the average of results from three measurements performed with methyl viologen (see “MV protein quantification” in Materials and Methods). chl, chlorophyll.

To test our software, we performed the R. capsulatus assay on 20 engineered strains of Chlamydomonas reinhardtii with known expression levels of a synthetic H_2_-producing enzyme (29). To produce images for the software, these strains were plated on four replica plates of 20 clones each. Two images were obtained for each plate: a colony image and a halo image of the GFP fluorescence. Fig. 3A shows a composite (manually colored and contrasted) image of one of the plates from this assay. We analyzed these images using CFQuant (Fig. 3B and C show the identified colonies and halos, respectively, of the plate displayed in Fig. 3A) and obtained the features (measured values) of each halo and colony. To control for colony size, we divided the halo feature data by the colony feature data. We then averaged the results of all four plates and searched for the single feature that best predicted the known protein abundance (calculated from three repetitions of active-protein quantification; see “MV protein quantification” in Materials and Methods). Of the 10 halo features, the best predictor was the pixel sum normalized by each halo's individual threshold (see Materials and Methods). This value (divided by the colony area value) had a Spearman's rho of 0.854 (*P* value < 10^−5^) when correlated with the known protein abundance levels (Fig. 3D). To further validate the performance of CFQuant, we performed a manual analysis of the same images using ImageJ (23). In measuring a similar normalized pixel sum divided by the colony area, the correlation of the values with the known protein abundance had a Spearman's rho of 0.759 (*P* value < 2*10^−4^) (Fig. S2). Levels of correlation of other values (area, sum, etc.) divided by colony area with the protein abundance were lower (not shown).

10.1128/mSphere.00676-18.2 FIG S2 Correlation between the values measured manually with ImageJ and the known levels of protein abundance (*P* value < 2*10^−4^). The manual measurement is the pixel sum of each halo (normalized by reducing the minimal value of the halo from every pixel) divided by the area of its colony. Note that the values are presented on a larger scale than the values in Fig. 3D, since CFQuant reduces 16-bit images to a lower bit size for faster processing. Download FIG S2, TIF file, 0.02 MB. Copyright © 2019 Dafni et al. 2019 Dafni et al. This content is distributed under the terms of the Creative Commons Attribution 4.0 International license.

## DISCUSSION

In this work, we developed CFQuant—a MATLAB-based image-processing tool built to meet the needs for a growing practice of using luminescent markers in large-scale assays. CFQuant is designed as a quick and reliable tool for quantifying colony luminescence while overcoming an array of prevalent forms of biological noise. CFQuant is a high-throughput tool. In addition to providing automatic analysis of individual plates, CFQuant also has a batch-processing mode that allows it to analyze multiple images with no user interaction. This enables CFQuant to provide fast analyses of very large numbers of colonies. These qualities allow CFQuant to be a considerably faster alternative to manual analysis of images, especially in working with larger assays.

### Halo edge detection.

The most fundamental issue that CFQuant had to overcome was the problem of automatically detecting halos. Most classical edge detection algorithms (such as are implemented in most generalist image analysis software) look for a noticeable drop in pixel intensity in order to determine object edges (30). However, colony luminescence halos tend to be gradual, with soft edges (see Fig. S3A in the supplemental material), and lack this drop in pixel intensity. In manually adjusting the contrast, it might appear as if clear edges are present (Fig. S3B), but the pixels hidden by the contrast settings would still pose a problem for automatic tools. Therefore, mainstream edge detection methods struggle to define clear halo boundaries (Fig. S3C). Permanently changing the image's pixel values to the manually selected contrast values could remedy this problem but would do so at the expense of loss of information from the image.

10.1128/mSphere.00676-18.3 FIG S3 Comparison of halo identification methods on a halo image. (A) The unedited image. The halos are slightly darker than the rest of the plate. (B) The image after contrast adjustment, with the halos now easy to spot. (C) The result of edge detection on the original image. The white lines represent the detected edges. Detection was carried out using the MATLAB edge function with the Canny method (30), a threshold value of 0.006, and a sigma value of 7. Parameters were gradually adjusted (first the threshold, then the sigma, and finally the threshold again) to maximize the edges around the halos while reducing noise. Other methods were also tested but performed more poorly than Canny (not shown). (D) The boundaries detected by CFQuant. Download FIG S3, TIF file, 0.9 MB. Copyright © 2019 Dafni et al. 2019 Dafni et al. This content is distributed under the terms of the Creative Commons Attribution 4.0 International license.

We tackled this issue in a different way; as CFQuant is a software program optimized for detecting and quantifying colony luminescence, we used some of the natural traits of these assays to solve the problems of halo detection. Instead of looking for steep decreases in pixel intensity, we exploited the fact that halos are roughly centered around the colony producing them—and thus, we begin our halo search from the colony coordinates. From this point, the area analyzed by the software gradually spreads out in a search for the halo’s edge. Since halation takes on a circular shape by definition, we can look for radial signals instead of intensity drops; thus, CFQuant is able to detect halo boundaries properly (Fig. S3D), uniformly and automatically—making it suitable for large-scale assays. It should be noted that the halo centers are not expected to be exactly at the location of the colony centers, and thus, our method allows for some freedom in their alignment (Fig. 3C).

This method also allows CFQuant to overcome another common issue in halo identification—overlaps. By identifying the partial circle belonging to each of the overlapping halos, CFQuant is able to estimate the original boundaries of each halo. These boundaries are later used to avoid overestimating the intensity of overlapping halos. CFQuant performs well even in assay plates with many overlaps (Fig. 3).

It is worth mentioning that in quantifying images of colony fluorescence from the R. capsulatus assay, we achieved better results with CFQuant than were achieved in measurement of the images manually with ImageJ (Spearman's rho of 0.854 versus 0.759, respectively, when correlated with the known protein abundance). We believe this is mostly due to CFQuant's ability to compensate for the halo overlaps, which were abundant in our images (Fig. 3C), though it is also possible that the determination of halo edges was less consistent when working manually.

### Using CFQuant.

In this work, we tested the performance of CFQuant on a specific assay and obtained high levels of correlation with active-protein abundance measurements in using the normalized pixel sum of the halos. However, CFQuant was built as a generic tool for colony assays that produce luminescent halos. It is unlikely that the same features of the colonies and halos would provide optimal measurements for every assay of this sort. For this reason, CFQuant calculates and returns multiple features on both the colonies and the halos.

When using CFQuant for the first time, we strongly encourage users to calibrate their assay. Users should start by quantifying the probed trait (e.g., protein abundance, as in the experiment we describe here) using a known method. In the next step, users should run the luminescence assay on the same colonies, analyze the results using CFQuant, and search for the value (or set of values) that best predicts the probed trait. We do expect that dividing halo feature data by colony feature data will improve correlations, as doing so normalizes for colony size. Depending on the assay used, there may be additional requirements, such as the use of a minimal or maximal colony size that can be analyzed reliably, etc. With the R. capsulatus assay, algae colonies need some time to grow before producing enough hydrogen to have visible fluorescence.

Another factor to consider is colony placement. While CFQuant can identify randomly scattered colonies, a few limitations do exist with respect to reliable halo identification and quantification. It is advised not to place colonies closer together than the expected radii of their halos. CFQuant handles partial overlaps between halos, but large overlaps can disrupt halo detection and prevent reliable quantification of halo values, particularly if multiple halos overlap and completely obscure the background. It is also advised to keep similar distances between the colonies and the edge of the plate; otherwise, the halos might be cut off by the edge, causing underestimation of their size.

## MATERIALS AND METHODS

### Software overview.

After uploading the colony and halo images, the user can select from two different methods of colony detection. If the colonies have a known arrangement, the user can input a simplified version of it (see “Image requirements” below) that CFQuant will use for arrangement-based detection. If not, the software can use scatter-based detection, where no previous assumptions on colony number or arrangement are made.

Either way, CFQuant begins automated identification of the colonies with a background elimination stage. In arrangement-based detection, that step is followed by image rotation (to ensure that the image aligns with the specified arrangement) and, finally, testing of foreground areas against the user-specified arrangement to delete any persisting background noise and join split colonies. In scatter-based detection, persisting noise is filtered out based on shape and pixel values, and detection of split colonies is not carried out. After the automated identification, the results are displayed and the user can correct mistakes in identification (if any). After user approval, data on the colonies are collected.

Next, the luminescent halos are automatically identified using the location of the colonies. The program searches for the largest area around each colony that is approximately circular. Incomplete circles are also accepted, to account for overlapping halos. After identification, the user is once again prompted to make any necessary corrections, and then the data on them are collected.

CFQuant can also perform batch processing, analyzing multiple images without user interaction.

A simplified flow chart of the image analysis stages is shown in Fig. 2.

### Image requirements.

The program requires one image of the colonies and a second image of the luminescent halos. The two images should be taken with the same camera, using the same focus and camera location, since the location of the colonies is used in analyzing the halo image. Additionally, the colonies must be the darkest or brightest objects in the image, and the same is also recommended for the halos (though areas outside the halos should not affect the analysis). Scatter-based colony detection can handle some noise from areas that are darker/brighter than the colonies, but it is recommended to avoid this if possible.

The images can be in any format supported by MATLAB, including TIF, BMP, GIF, PNG, and JPEG. The images can have any bit depth, but it is recommended to use 16-bit images if possible. Such images are reduced by CFQuant to have values of only between 0 and 511 (effectively 9-bit, though stored as 16-bit) for faster analysis, but they still have a larger value range and thus provide more-accurate results.

In arrangement-based colony detection, the colonies need to be arranged on a grid of rows and columns (similar to those shown in Fig. 2B and 3A). The arrangement does not need to be precise, but rows and columns should be roughly recognizable. This arrangement is specified by the user upon initiation of the process and is used for the automatic identification of the colonies.

### Colony identification.

CFQuant has two methods of colony detection: arrangement-based and scatter-based detection. Arrangement-based detection uses a simplified arrangement (input by the user) of the colonies to identify the colonies more reliably and to detect split colonies and join them. Scatter-based detection requires no user input aside from the images; however, it is more error prone and cannot join split colonies.

After the colony image has been loaded (Fig. 2A), both methods begin by calculating a minimal estimate of the background area in the image, under the assumption that the distance between adjacent colonies is no smaller than the average diameter of the colonies. In arrangement-based detection, it is calculated from the user-specified arrangement as follows:

(1) $$A_{B}=\frac{A_{T}N\pi R^{2}}{2R\left(2r_{n}-1\right)2R\left(2c_{n}-1\right)}=\frac{A_{T}N\pi }{16\left(r_{n}-0.5\right)\left(c_{n}-0.5\right)}$$

where *A_B_* is the background area estimate, *A_T_* is the total image area, *N* is the number of colonies, *R* is the average radius of a colony, and *r_n_* and *c_n_* are the numbers of row and columns, respectively, in the user-specified arrangement.

In scatter-based detection, the colony number and arrangement are both unknown, so a modified equation is used:

(2) $$A_{B}=\underset{N\to \infty }{\lim}\frac{A_{T}N\pi }{16\left(1-0.5\right)\left(N-0.5\right)}=\frac{A_{T}\pi }{8}$$

where *r_n_* was set to 1, *c_n_* was set to *N*, and *N* approaches infinity, thus decreasing the calculated area to a minimum.

This value is used to ensure that the colonies have higher pixel values than the background. Background values are expected to have a considerably smaller range than the colony values. Therefore, the software compares the range of low pixel values in the image that occupy the estimated background area to the range of high pixel values that occupy the same area. If the range of high pixel values is smaller, the image values are reversed.

CFQuant then sets an initial background threshold value (i.e., the highest value belonging to the background) as the highest value under which the background area is still no larger than the minimal background area estimate.

This initial background threshold might not eliminate enough of the background, and some colonies might remain connected in the same foreground area. Therefore, CFQuant tests the entirety of the foreground area to see if any of them can be split by some increase to the threshold value. To guarantee that the split is between two colonies, it is accepted only if both parts are still in the foreground after a second, identical increase. The highest value to cause such a split (if any) is selected as the new threshold value.

From this point, the two detection methods differ greatly. In arrangement-based detection, small areas of persisting background noise is deleted by the following criteria:

(3) $$A_{\text{Delete}}<\frac{A_{n}}{5}$$

where *A*_Delete_ is of a size small enough to delete and *A_n_* is the size of the *n*th largest area, with *n* being the number of colonies in the user-specified arrangement. The use of the 5 value is based on parameter optimization.

This concludes the background removal process (Fig. 2B). However, excess foreground areas that remain after this stage can still represent either persisting background noise or split colonies or both. To determine how many of them represent background noise, CFQuant creates multiple copies of the colony image, with each copy representing a possible solution to the classification problem. One copy uses the selected background threshold value, but other copies use increasingly larger threshold values to delete excess areas. The excess areas remaining in these copies are regarded as parts of split colonies and are joined on the basis of proximity until no excess areas remain.

The software then ensures that the image is not rotated (i.e., that the colony rows are horizontal). In each copy image, the distances between colony canters (after joining) and their corresponding angles are calculated and are normalized to be within ±45°. The software then pools the same number of shortest distances as the number of distances between adjacent colonies in the user-specified arrangement. After removal of outliers in distance and angle, the average angle is used as the rotation angle of the image, which is then rotated accordingly.

Following the rotation, the colony centers are used to calculate the ideal location of each colony on the basis of the locations of the centers and the average distances between them. Each colony is then assigned to an ideal location based on proximity and position. The sum of the squares of the distances between the colonies and their ideal location is then used to evaluate each copy image, and the one that resulted in the smallest sum is chosen.

In scatter-based detection, these stages do not exist, and persisting areas of background noise are deleted based on their shape and values. To use those reliably, CFQuant first tests if any 1 of the 10 largest foreground areas is comprised mostly of pixels with the same small value. If so, that value likely still represents part of the background, and the threshold value is raised to erase it.

Following this step, all colonies are expected to have a roughly circular shape, so the circularity of each foreground area is tested using the following equation:

(4) $$C=\frac{A}{\pi R_{m}^{2}}$$

where *C* is the circularity value, *A* is the area, and *R_m_* is the distance from the area's center to its furthest edge point. Colonies with a circularity value below 0.18 are treated as background and deleted. This value is based on parameter optimization and is about half of the lowest circularity value observed for any colony in our plates.

After the circularity test, CFQuant once again tries to increase the background threshold value. For every possible threshold, the software compares the foreground areas that are to be deleted by it with those that are not to be deleted by dividing the smallest mean value of the persisting areas by the largest mean value of the deleted ones. If the new threshold separates high-value colonies from low-value noise, that division value is expected to be larger than 1, and so the threshold with the largest division value above 1.2 is selected (value based on parameter optimization). If no such threshold value is found, the old threshold value is kept.

As a final step to delete background noise, the foreground areas are sorted into groups based on their sum, with the smallest sum in the first group being double the largest sum of the next group and so on. If this creates two or more groups, the software tests if a group exists that has the largest total sum and the largest number of areas. If such a group is identified, only that group is selected. If not, the groups are sorted on the basis of the maximal pixel values of their foreground areas. The group with the largest maximal value is selected, as well as any group whose maximal value is higher than the selected group's average maximal value. Areas that belong to groups that were not selected are deleted, based on the assumption that colonies have much higher sums than background noise. This also allows the deletion of large areas such as smears on the plate.

Once all colonies are identified (regardless of method), CFQuant identifies the minimal splitting value (i.e., the lowest value separating the colonies into different foreground areas) and the highest value where no colonies is deleted. The background threshold value used for data acquisition is set as the mean of these two values. This value seems to fit well with the visible borders of the colonies (Fig. 2C).

After this stage is complete, CFQuant displays an interface that enables manual deletion and addition of whole colonies or split parts, if necessary.

Once the user confirms the identification, data on the colonies are calculated (see “Measured values” below).

### Halo identification.

In images from our laboratory, the halos often have “holes” with lower values at the location of the colonies (Fig. 2D). CFQuant begins halo identification by filling these low-value areas, using the following criteria to find them:

(5) $$D_{\text{min}}<R\text{ and }D_{\text{max}}<2R$$

where *D*_min_ is the distance between the colony center and the point on the area that is closest to it, *D*_max_ is the distance between the colony center and the point on the area that is furthest from it, and *R* is the mean radius of the colonies. The multipliers for the colony radius were chosen by parameter optimization.

Next, CFQuant makes sure that the halos have higher values than the background. To do so, the software repeats the hole-filling process for both the regular image and the image with reversed values. It then determines the number of pixels that have higher values than the colony centers in each filled image. If the regular image has more of these pixels, it means that the background has higher values than the halos, and the reversed image is used instead.

After these initial corrections to the image, CFQuant starts inspecting each halo individually. To account for noise in the location of the halos' centers, their potential centers are set as the center of the halo's colony, as well as any point up to 1.5 times the mean radius of the colonies away from that center (specific value chosen after optimization). Next, the software attempts to fit a background threshold value to each halo.

Starting from the highest threshold value possible, CFQuant finds the foreground area containing the colony center, and the boundaries of that area, ignoring inner boundaries with areas smaller than the average colony area and boundaries caused by the edge of the image. For each possible center location, the halo radius is calculated as follows:

(6) $$R=R_{\text{min}}+\text{ max}\left(1,0.045\text{*}D-0.2\right)$$

where *R* is the selected radius and *R*_min_ is the distance from the specific center location to the closest boundary point. In cases in which the colony arrangement was specified, *D* is the mean of the average row distance and average column distance. Otherwise, it is the average distance between adjacent colonies. It is used as a scale for the expected size of the halos. The constants in this equation were optimized on multiple images (see “Parameter optimization” below).

Once the halo's radius is found, a circularity test is performed. The algorithm checks which border points are within that distance from the center, and these border points are translated to angle coverage. If the border points form arcs with a total angle of at least $\frac{4}{5}\pi$ (in radians), the halo is accepted for that threshold (the specific value was optimized; see “Parameter optimization” below). Accepting incomplete circles like this is necessary, since overlapping halos do not form a full circle (Fig. 1D).

This circularity test is done with every possible center. If the halo passes the circularity test, the center that results in the highest radius (while still passing the test) is selected, and the software proceeds to test the halo with a lower threshold value (and thus with a larger area). If not, the software assumes that the halo ended at a higher threshold value. However, the software still reduces the threshold value and performs the test again as long as any other halo is still circular. This means a halo could fail the circularity test due to some noise and then pass it with a lower threshold value.

Once a threshold value is set for all the halos, the results are presented to the user. The user can then shrink or expand a halo (which changes its threshold value), delete one entirely, or create a halo for a colony with no identified halo.

Once the user confirms the identification, data on the halos are calculated (see “Measured values” below).

### Measured values.

CFQuant calculates multiple parameters for each colony or halo, including the total area in pixels as well as the sum, mean, and maximal pixel values.

Additionally, two sets of normalized values are calculated for each of the sum, mean, and maximal pixel values by subtracting a different threshold value from each pixel of the colonies/halos prior to the calculation. For colonies, the selected background threshold and the minimal splitting value are used (see “Colony identification” above). For halos, the individual threshold of each halo and the lowest of these individual thresholds are used (see “Halo identification” above).

For the halo image, pixel values in areas of overlaps of halos are calculated differently, since the combination of the influences of the two halos can artifactually increase pixel values (Fig. 1D). CFQuant relies on the calculated radius of the halos at every threshold value. For each halo, each pixel in the overlapping area gets the value corresponding to the highest threshold where it is still within the radius of that halo.

### MV protein quantification.

Protein quantification was carried out precisely as previously reported (26). Briefly, following 2 h of dark anaerobiosis, cells were transferred into a buffer containing reduced methyl viologen and Triton X for lysing the cells. A 500‐μl sample was drawn from the headspace, and the H_2_ concentration was determined by gas chromatography. The amount of enzyme was calculated based on the constant enzyme’s specific activity.

### Rhodobacter capsulatus high-throughput screen.

A high-throughput screen was done using previously described methods (26). Algal strains, overlaid with engineered H_2_-sensing R. capsulatus, were scanned using a Fuji FLA-5100 fluorescence imager. A 473-nm-wavelength laser was used for excitation, whereas 510-nm and 665-nm filters were used for quantifying emerald GFP luminescence and chlorophyll density, respectively.

### ImageJ manual quantification.

Analysis was performed on ImageJ 1.51j8, using the same images as were used in the performance test (see Results).

Colony area was calculated by applying a threshold value to the colony image and then using the “analyze particles” option.

Halo data was collected for each halo individually. A perfect circle was drawn around each halo, and then the “measure” option was used, yielding the area, mean value, and minimal value of the halo. The normalized sum of the halo was then calculated as follows:

(7) $$S_{\text{norm}}=A\left(\bar{V}-V_{\text{min}}\right)$$

where *S*_norm_ is the normalized sum, *A* is the area, *V̄* is the mean value, and *V*_min_ is the minimal value. This is comparable to the normalized sum calculation from CFQuant, with *V*_min_ substituting for the halo threshold value.

Finally, this value was divided by the colony area for comparison with the known protein values of each colony.

### Parameter optimization.

To optimize the parameters of CFQuant, we ran the software on a set of 38 image pairs (colony and halo) of the R. capsulatus assay. The software was used multiple times with different values for each parameter individually, and the automatic identification results were manually inspected. The value that was selected was the one that resulted in the best fit with the perceived boundaries of the colonies or halos. Seven of the images had multiple (up to 656) scattered colonies and were not used to test arrangement-based detection.

All manual comparisons between the automated CFQuant results and the perceived colonies or halos in the images were performed by the same member of the team, using the same computer each time (desktop computer running Windows 7).

### Data availability.

CFQuant is available at https://www.energylabtau.com/cfquant.
