# Supplementary figures and images for: Image-Processing Software for High-Throughput Quantification of Colony Luminescence
Source: mSphere. 2019 Jan 2;4(1):e00676-18. doi: 10.1128/mSphere.00676-18 (PMC6315083; doi:10.1128/mSphere.00676-18)

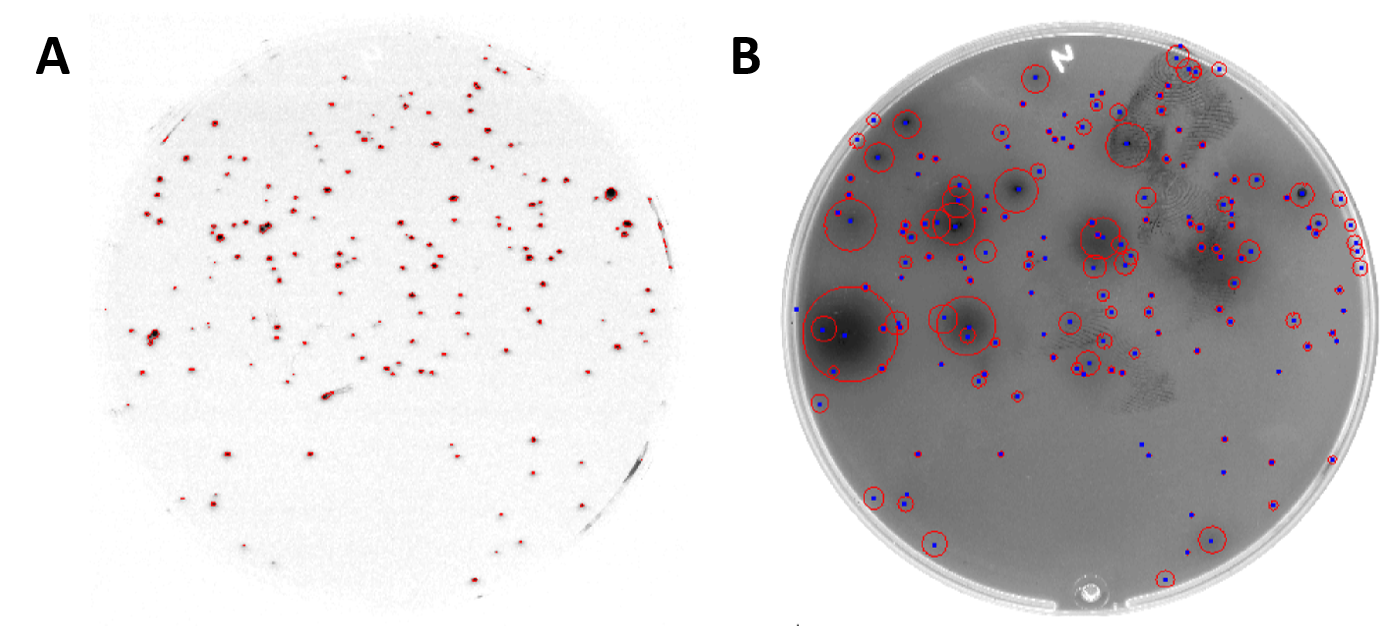

Supplement: FIG S1 [file sph001192734sf1.tif]

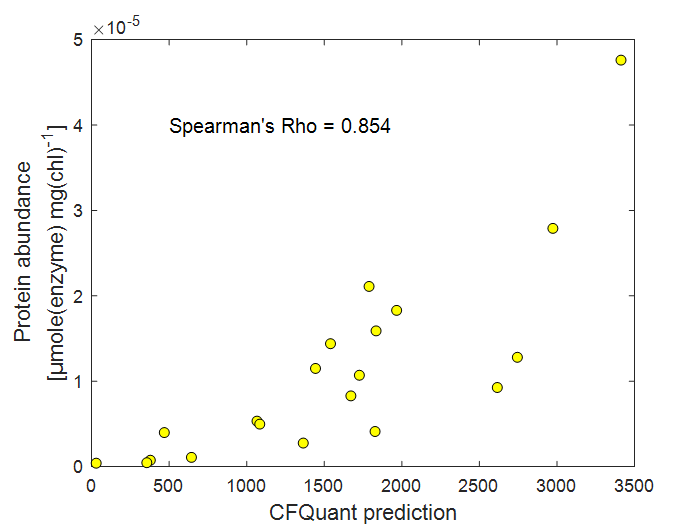

Supplement: FIG S2 [file sph001192734sf2.tif]

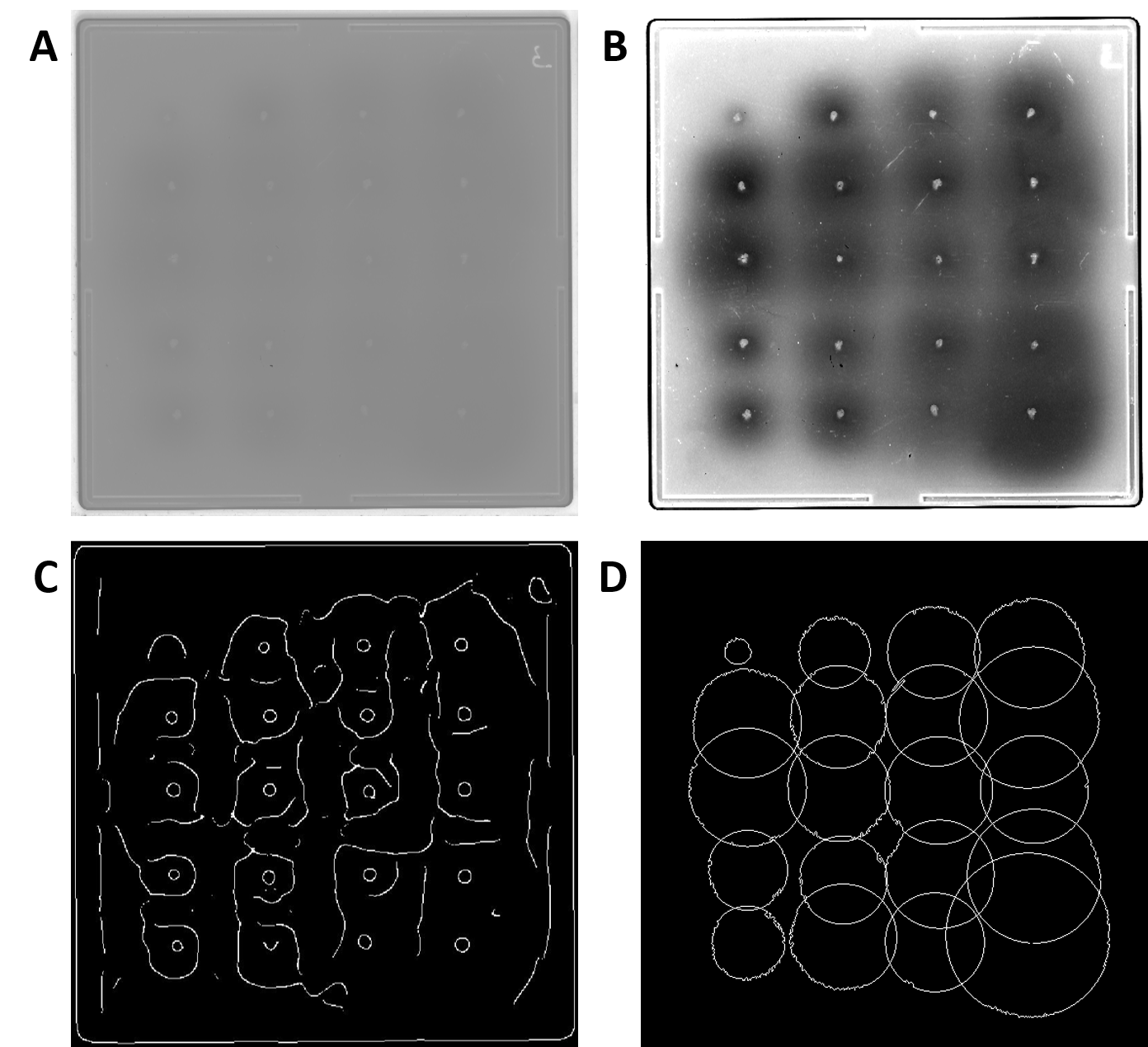

Supplement: FIG S3 [file sph001192734sf3.tif]
